# “I want to lift my people up”: Exploring the psychological correlates of racial themes within the life stories of midlife Black Americans

**DOI:** 10.1111/jopy.12932

**Published:** 2024-04-12

**Authors:** Ananya Mayukha, Ambar Guzman, Sirin Jitklongsub, Dan P. McAdams

**Affiliations:** ^1^ Department of Psychology Northwestern University Evanston Illinois USA

**Keywords:** generativity, narrative identity, race

## Abstract

**Objective:**

This study explores how middle‐aged Black Americans talk about race, without prompting, while telling their life stories.

**Method:**

Drawing upon a dataset of lengthy Life Story Interviews (*N* = 70), we first employed a keyword search to identify race‐relevant interview scenes for each participant. Next, we conducted a thematic analysis of these scenes to identify salient racial narrative themes. Finally, we coded race‐relevant scenes to examine the psychological correlates of racial narrative themes.

**Results:**

We identified 460 total racially themed Life Story Interview scenes, with the number of racially themed scenes ranging from 1 to 17 across participants' interviews. Racial narrative themes included *Community of Care*, *Black Cultural Identity*, *Multiculturalism*, *Activism*, *Encounter with Racism*, *Systemic Racism*, and *Racial Reckoning.* Quantitative analyses highlight a relationship between racial narrative themes and psychological measures of wisdom and generativity.

**Conclusion:**

This study offers insight into the ways that race manifests in the life stories of Black Americans and highlights the importance of considering race in the study of narrative identity, and personality, more broadly.

## 1 INTRODUCTION

Human beings make sense of their lives through stories. Personality and developmental psychologists use the term *narrative identity* to refer to an integrative and evolving story in the mind of a person that reconstructs the past and imagines the future (McAdams, 2001; McAdams & McLean, 2013; Singer, 2004). The story addresses questions like this: *Who am I? Who am I becoming? What does my life mean?* (McAdams, 2021). While this story develops internally, it is permeable to the outside world: cultural context shapes how people construct their stories, and stories in turn position individuals as active participants in society as they make sense of who they are, who they are becoming, and what their lives may mean (Hammack, 2008; McLean & Syed, 2015).

The current study explores how one culturally defined construct, race, is reflected in the narrative identities of middle‐aged Black Americans who grew up during the civil rights era. This study bridges the long history of research on racial identity with the narrative identity framework to consider how race may function within a person's broader story. In turn, we explore how the tendency to incorporate race into one's life story relates to three markers of psychological health at midlife: well‐being, wisdom, and generativity.

### 1.1 Race and identity

U.S. history is steeped in notions of white supremacy and anti‐Blackness, which continue to shape the present‐day American cultural context (Rogers, Niwa, et al., 2021).  Racial groups were originally devised to establish a hierarchy of humanness that reinforced these ideologies, with those categorized as “white” at the top, those categorized as “Black” at the bottom, and all other groups judged by proximity to these anchors (Rogers, Niwa, et al., 2021). While the implications of racial categorization have shifted through acts of resistance across history, racism persists, and continues to shape the daily experiences—and identities—of Americans. Amidst this historical context, psychologists have long been interested in how individuals navigate racism and develop a sense of racial identity.

Theories of racial identity development consider both *process—*how individuals' attitudes toward their racial group change over time—and *content—*what their attitudes are (Galliher et al., 2017; Scottham et al., 2010). Cross's (1971) Nigrescence theory offered an influential framework for understanding the process by which Black Americans navigate their racial identities. Initially unaware of race (*Preencounter*), Cross (1971) suggested, Black individuals are faced with an experience (*Encounter*) that prompts them to grapple with their new racial consciousness (*Immersion‐Emersion*), which allows their racial identity to take hold and deepen (*Internalization*), ultimately contributing to a sense of collective identity and motivating activism (*Internalization Commitment*) (Neville & Cross, 2017). The theory cautioned that not all individuals go through every stage of this process, and individuals may also cycle through different stages at different times in their lives.

On the content side, Sellers et al.'s (1998) Multidimensional Model of Racial Identity (MMRI) sets forth four dimensions of racial identity—racial salience, racial centrality, racial regard, and racial ideology—on which individuals naturally differ. The dominant approach to research on racial identity has been to translate these dimensions into quantitative measures and to examine relationships between these measures of racial identity and psychological outcomes. For example, the *racial centrality* dimension of the MMRI, which measures how important race is to a person's self‐concept (Sellers et al., 1998), has shown correlations with achievement motivation among African American girls (Butler‐Barnes et al., 2018), civic engagement and activism among Black young adults (Chapman‐Hilliard et al., 2022; Christophe et al., 2022; Pender et al., 2019), and healthcare mistrust among African American adults (Cuevas & O'Brien, 2019). Furthermore, while people higher in racial centrality tend to report more experiences of discrimination (Burrow & Ong, 2010; Chae et al., 2017; Sellers & Shelton, 2003), racial centrality also serves as a protective factor against the negative impact of these experiences (Seaton & Zeiders, 2021; Sellers et al., 2003).

Whereas these studies shed light on the psychological significance of racial identity, a growing body of qualitative studies have probed into people's lived experiences as members of their racial group. These studies are broad‐ranging and include topics such as how children's racial identity narratives changed with the surge of the Black Lives Matter movement (Rogers, Rosario, et al., 2021), how African international students make sense of their Black racial identity in the United States (Asante et al., 2016), and how African American elders reminisce about their life experiences as African Americans (Shellman, 2004). A common approach across most qualitative studies of racial identity is to directly prompt individuals to talk about their racial experiences. The current study takes a different approach, exploring race from the vantage point of a person's broader narrative identity. While a focus on narrative identity does not preclude direct prompting about a person's racial experience—and indeed, racial identity could be conceived as part of a person's narrative identity—studies of narrative identity have not historically included prompts about race. Drawing on data from one of the most extensive studies of narrative identity to date, we consider how *even without prompting*, a sample of Black Americans talk about race while telling their life stories.

### 1.2 Narrative identity

Considering race from the vantage point of narrative identity, we draw on Erik Erikson's (1963) conception of identity as an arrangement of the self, typically achieved in young adulthood but evolving thereafter, that situates the person in the adult world while providing a person's life with a sense of “inner sameness and continuity” (p. 261). Erikson (1963) characterized the *awareness* of identity as a process of reckoning with discontinuity, both within oneself and with respect to one's positioning in the broader social world.

Building on Erikson's conception, McAdams (1985) suggested that the problem of identity first arises when adolescents and young adults sense discontinuity and inconsistency in their lives, across roles and over time, and seek to resolve the problem through narrative. The discontinuity may arise in many different forms. For example, a young person may believe that “when I am with my friends, I am outgoing and speak my mind, but when I am in class, I am quiet and don't say a word”; or, “when I was younger, I thought all white people were bad but now I host people from all over the world regularly at my home.” In order to make sense of such varied and conflicting experiences, individuals construct stories to explain how they have changed over time and what the different roles in their lives may mean. These stories comprise a person's narrative identity (McAdams & McLean, 2013; McLean et al., 2007), which develops as an internal, continually evolving narrative that integrates a person's experiences together into a coherent sense of self. In this way, narrative identity serves as a psychological resource, providing individuals with a sense of unity and purpose in their lives (McAdams, 2021). McAdams (1995) further conceived of narrative identity as one of three layers of personality, with the first layer consisting of a person's dispositional traits, the second layer consisting of a person's characteristic motivational agenda, and the third layer consisting of a person's identity and evolving life story. By exploring the racial content of life stories, we thus highlight the relevance of race to the study of personality among Black Americans, at the level of narrative identity.

### 1.3 Narrative identity and culture

Whereas identity is in one sense deeply personal, it is also profoundly cultural. Erikson (1968) described identity as a process that takes place, “in the core of an individual and yet also in the core of his communal culture, a process which establishes, in fact, the identity of those two identities” (p. 22, as cited in Rogers, 2018). Likewise, McAdams (2021) describes narrative identity as a psychosocial construction, co‐authored by individuals and their surrounding cultures.

Methodological approaches for examining how narrative identity interacts with culture typically rely on the analysis of life stories. Methods include comparing narrative themes across cultural groups (e.g., Boytos et al., 2020; Reese et al., 2014, 2017; Turner, 2022; Wang & Singer, 2021), examining how personal narratives align with and deviate from broader cultural scripts or master narratives (e.g., Hammack, 2010; McLean et al., 2018; Westberg, 2022 ), exploring life narratives of a nondominant cultural group (e.g., Bigabo & Jansen, 2020; Ramirez & Hammack, 2014), and eliciting narratives about cultural events or experiences (e.g., Weststrate & McLean, 2010).

Although these studies differ in their specific approaches, one common methodological feature is that they involve collecting narrative accounts of specific experiences, events or memories, and evaluating a standard set of narratives for each participant (Adler et al., 2017). For example, Weststrate and McLean (2010) asked 251 participants to describe (1) a cultural event and (2) a self‐defining memory that shaped their gay or lesbian identity, and their subsequent analysis considered features of participants' responses to these two prompts. Likewise, Reese et al. (2014) conducted interviews with 268 adolescents, coding three interview scenes—(1) high point, (2) low point, and (3) turning point narratives—for narrative themes and comparing themes across cultural groups.

The current study explores a different approach. Considering the culturally defined construct of race, we probe a set of Life Story Interviews to identify the specific places within each interview where our participants, Black Americans who grew up in the civil rights era, talk about race. While an analogous study might examine how participants talk about race when prompted and within the description of a particular kind of event, we explore how race comes up without prompting. Furthermore, we consider that individuals may differ in terms of *where* in the interview they bring up race, and we first attempt to capture this unique subset of “racial narratives” for each participant. This approach allows for a closer investigation of race within narrative identity, while also capturing differences in the extent to which individuals spontaneously incorporate race into their life stories.

### 1.4 Psychological correlates of narrative identity

In addition to identifying race‐relevant scenes and probing these scenes for salient racial narrative themes, we explore the potential psychological relations of these themes. Considering the developmental context of our participants, we explore relations between racial narrative themes and three measures of psychological health at midlife: well‐being, wisdom, and generativity.

Well‐being, which refers to a person's overall sense of meaning and purpose in life, is considered an important outcome measure across psychological disciplines. The narrative identity literature reveals a clear link between narrative features and psychological well‐being. Adler et al. (2016) delineated this link across four dimensions of narrative identity—motivational themes such as agency and communion, affective themes such as positive and negative emotional tone, meaning‐making themes such as growth and exploration, and structural elements such as narrative coherence—noting where narrative variables add incremental validity as predictors of well‐being compared to other variables across a number of studies (e.g., Adler, 2012; King et al., 2000; McAdams et al., 2001; Reese et al., 2011). Likewise, a substantial body of research has explored links between dimensions of racial and ethnic identity and positive mental health outcomes (e.g., Chew, 2015; Neblett et al., 2012; Rivas‐Drake et al., 2014; Smith & Silva, 2011; Yip et al., 2019). For example, in a meta‐analysis of studies utilizing the Racial Identity Attitudes Scale (RIAS) and various measures of psychological well‐being, Chew (2015) reported a positive correlation between the Internalization subscale of the RIAS, which measures the extent to which race is positively incorporated into a person's self‐concept, and psychological well‐being. Bridging this literature, the current study explores how the presence of racial themes within narrative identity may correlate with psychological well‐being.

Importantly, much of the existing research on racial and ethnic identity and positive mental health outcomes focuses on younger samples (Chew, 2015; Smith & Silva, 2011). Given the age group of our sample, we explore two additional developmentally appropriate psychological outcomes: generativity and wisdom. Within Erikson's (1963) theory of psychosocial development, generativity represents the central challenge of middle adulthood, as the core question guiding a person's development shifts from, “Who am I?” to “What will I leave behind?” (McAdams, 2018). Generativity reflects a person's commitment to caring for future generations through activities such as parenting, teaching, mentoring, leadership, and service to the community (McAdams, 2018). McAdams and de St Aubin (1992) lay out how generativity ultimately finds expression through narrative identity: a combination of cultural influences and inner desire prompt a person to develop a sense of concern for future generations, which ideally leads to a deeper commitment to developing generative goals, which in turn promotes generative *action*. Examples of generative action include giving birth to children, coming up with creative ideas, preserving rituals and traditions, participating in social movements, and letting go of one's generative efforts in the hope that what was created and nurtured will continue to flourish (McAdams, 2018). Throughout this process, individuals make narrative sense of their experiences, telling stories about their generative concerns and actions that are ultimately incorporated into their broader life stories.

Likewise, later adulthood is characterized by the challenge of accepting one's life experiences as, “something that had to be” (Erikson, 1963, p. 268), a process that nurtures a growing sense of wisdom (Erikson, 1982). While there is debate among psychologists about what, exactly, constitutes wisdom, a recent meta‐analysis identified two general approaches to conceptualizing wisdom: *performative wisdom*, which captures how individuals respond in specific scenarios, and *phenomenological wisdom*, which captures people's recollections of their daily internal experiences (Dong et al., 2023). Dong et al. (2023) suggest that differences among measures have resulted in ambiguities in the nomological net of wisdom. For example, although meta‐analytic results suggest a statistically significant correlation between wisdom and age, this correlation is moderated by wisdom type and wisdom measure, with most wisdom measures showing no significant relationship with age (Dong et al., 2023). That said, psychologists generally agree that wisdom reflects a sense of psychological maturity, gained through life experience, that allows certain older people to guide and counsel younger generations (McAdams et al., 2022; Taylor et al., 2011). Previous research has suggested that the ability to guide and counsel younger generations may be especially important among Black Americans: Black Americans tend to score higher on measures of generativity than white Americans (e.g., Hart et al., 2001; Newton & Baltys, 2014) and are also more likely to see themselves as sources of wisdom for their children (McAdams, 2013). The current study builds upon these findings to consider how the tendency to incorporate race into one's life story may relate to generativity and wisdom.

### 1.5 Research aims

The central aim of the current study is to explore how race manifests in the life stories of Black Americans. To do this, we employ a sequential mixed methods design^1^ with three phases: first, we identify race‐relevant scenes and explore the prevalence of these scenes within a larger set of life story interviews. Second, we inductively derive a set of “racial narrative themes” from these scenes. Third, we code each scene for the presence or absence of these themes to examine correlations between racial narrative themes and three psychological outcomes: psychological well‐being, wisdom, and generativity. It is worth highlighting that most existing research on identity is conducted with white, young adult, and emerging adult college students (Mitchell et al., 2021), and our focus on middle‐aged Black Americans expands this literature. Moreover, our multi‐method approach considers not only the racial content of narrative identity but also how this content corresponds with psychological outcomes. Our inductive qualitative analysis is thus strengthened by an exploratory, data‐driven approach, and the qualitative and quantitative nature of this study allows for a broad exploration of how race permeates the narrative identities and psychological health of middle‐aged Black Americans.

## 2 METHODS

### 2.1 Participants and procedure

Participants for this study were drawn from the larger Foley Longitudinal Study of Adulthood (FLSA; e.g., McAdams & Guo, 2015), conducted at Northwestern University between 2008 and 2018. The original study was approved by the Northwestern University Office for Research Institutional Review Board (IRB) under the protocol ID STU00001801. Participants were recruited via public venues such as the internet, bulletin boards, and newspaper ads, in the city of Chicago and surrounding suburbs. The original sample included 163 Black (*N* = 73) and white (*N* = 90) late‐midlife adults. From this sample, 3 Black participants did not complete a Life Story Interview during Year 1 of FLSA and were thus excluded from the current study. In this study, 70 participants were included on the basis that they (1) completed a Year 1 Life Story Interview and (2) selected their race/ethnicity as “African American,” (*N* = 67), “Interracial” (*N* = 1), or “Other” (*N* = 2).^2^ Participants ranged in age from 52 to 56 years (*M* = 54.44, *SD* = 1.11) and were 73% female and 27% male. Annual family income levels of participants at the onset of the study ranged from under $25,000 to over $300,000, with a median income range of $50,000–$75,000. Education levels ranged from “high school” to “graduate work,” with a median education level of “college.”

Participants completed the Year 1 Life Story Interview (LSI; McAdams, 2008) in a dedicated interview room at Northwestern University. Of the nine trained interviewers—graduate students, postdocs, and faculty members—eight were white and one was Black and biracial (Reischer et al., 2021). In addition to the interview, participants also completed a battery of self‐report measures. Participants were compensated $75 for each survey battery and $75 for each interview completed. Interviews were audio‐recorded and professionally transcribed. Participants completed this process during Year 1, Year 5, and Year 9 of the study, with abridged versions of this protocol conducted in the years between. For the current study, we focus on data from Year 1 of FLSA.

### 2.2 The Life Story Interview

The LSI (McAdams, 2008) is a comprehensive set of 22 prompts or “scenes” designed to capture narrative identity. The Year 1 interview included seven sections, each containing 1–8 scenes: (1) *Life Chapters (1 scene)*, in which participants were asked think of their lives as a book or novel and summarize each chapter; (2) *Key Scenes (8 scenes)*, in which participants were asked to describe a high point, low point, turning point, positive childhood memory, negative childhood memory, vivid adult memory, religious, spiritual or mystical experience, and wisdom event in their lives; (3) *Future Script (3 scenes)*, in which participants were asked to describe the next chapter in their lives, their dreams, hopes and plans, and a life project they are currently working on; (4) *Challenges (4 scenes)*, in which participants were asked to describe their greatest life challenge, a health challenge, a loss, and a failure or regret; (5) *Personal Ideology (4 scenes)*, in which participants were asked about their religious, ethical, political, social and other values; (6) *Life Theme (1 scene)*, in which participants were asked to look back over their life and discern a central theme, message or idea; and (7) *Reflection (1 scene)*, in which participants were asked to share any reflections on the experience of the interview (McAdams, 2008). Importantly, the interview did not include questions about race. The full interview protocol is available here: https://osf.io/tr7zv/.

### 2.3 Context for current analysis

Although noted above, we wish to highlight several elements of the original study design which set parameters for the current investigation of racial narratives within FLSA. First, all but one interviewer was white. While narrative identity represents an internal story, sharing this story in the context of an interview is ultimately an interpersonal process. Listeners influence the stories people choose to relate to as well as the way people tell these stories (Pasupathi & Billitteri, 2015). For example, Rogers, Versey and Cielto (2021) offer a powerful example of how interviewers' shared positionality with their participants as Black women interviewing Black girls allowed participants to assume a “relational ‘we‐ness’” (p. 7) with their interviewers, shaping the way they shared their stories. In the case of the current investigation, it is important to recognize that subsequent findings largely reflect how participants talk about race while telling their life stories *to a white person*.

Second, none of the LSI questions directly asks participants to talk about race. While the Life Story Interview was designed to elicit a person's broader narrative identity, this does not preclude incorporating questions related to race. Racial identity scholars have highlighted how the content and wording of questions influence the extent to which participants engage with cultural constructs throughout an interview (Rogers et al., 2022). Thus, it is important to recognize that subsequent findings reflect how participants talk about race *without prompting* and that a different set of prompts might elicit a different conceptualization of the role of race within narrative identity.

Third, the generational group of participants—baby boomers born between 1951 and 1955—suggests that participants came of age during the Civil Rights movement. Our geographic sampling within the greater Chicago area further suggests that several participants were part of the Great Migration, having moved with their families from the South (e.g., Mississippi, Alabama) to the Midwest during their early childhoods. Likewise, Year 1 interviews were conducted between 2008 and 2010, in the months directly preceding and following the election of President Barack Obama, a time of apparent racial progress in the United States. We highlight these events to emphasize that subsequent findings should be considered with the particular historical context of participants' lives in mind.

### 2.4 Researcher positionality

In addition to the historical context shaping participants' lives, the current analysis, conducted between 2021 and 2023, was motivated and shaped by the historical context of researchers' lives: our analysis comes at a time of increased attention to racial justice following highly publicized violence against Black lives, the resurgence of the Black Lives Matter movement, and the widespread reckoning with the persistence of racism across time (Neville & Cross, 2017). Researchers' individual experiences also shaped their interpretations of participants' stories. Experiences shaping the first author's perspective included being a Southeast Asian (Indian) American woman in her late twenties, having parents who are immigrants, and working at a nonprofit where she organized community dialogues centered around race. Experiences shaping the second author's perspective included being a first‐generation, young Black woman in her early twenties, raised by Dominican family members in the Bronx. As a Psychology and Black Studies student, the second author drew upon both disciplines, along with her own national and diasporic identity to listen for the breadth of Blackness within participants' stories. The third author's perspective was shaped by her experience being a Southeast Asian (Thai) woman in her early 20s and by her earliest exposure to American culture—living temporarily in a predominantly Black and Mexican community in the United States as a child—as well as by conversations about race after returning to America as an adult. Experiences shaping the fourth author's perspective include being a white man in his late 60s who grew up in a segregated midwestern city, where race relations were fraught, as well as engaging in previous research and writing about narrative identity among Black Americans in midlife (e.g., McAdams, 2013).

### 2.5 Deriving racial narratives

From the full Life Story Interviews, we sought to identify the unique subset of scenes, for each participant, containing race‐related themes. Our focus on scenes aligns with existing guidelines for analyzing narrative data (e.g., Adler et al., 2017); however, our procedure for data preparation departs from previous studies of narrative identity in that we selected scenes for analysis based on the presence of specific thematic content. In particular, we conducted a keyword search for race‐related terms, with keywords derived from the data, and included only those scenes containing race‐related terms. “Race‐related terms” were defined as any words that the first three authors identified as connected to race in some way, whether explicitly or implicitly. Implicit references were included with the understanding that race permeates many aspects of a person's experience, and racism intersects with other systems of oppression such as classism and sexism (Cole, 2009; Crenshaw, 1991). The first author began by reading 10 full interviews, randomly selected from the data set, and keeping a list of any race‐relevant terms she could identify within these interviews. The second and third authors repeated this process with five additional interviews each.

Authors' keyword lists were merged, alphabetized, and edited to remove duplicates. Using the NVivo Text Search tool, each word was then assessed by examining the contexts in which it appeared in the dataset. Primary reasons for exclusion were that the word was too broad, turning up many instances that did not clearly relate to race (e.g., “attractive,” “background,” “bible”), or that the word occurred infrequently, without a clear connection to race. Examples of keywords in the final list are presented in Table 1 (organized by general category). The full list of keywords can be found here: https://osf.io/tr7zv/.

**TABLE 1:** Examples of keywords used to identify race‐relevant LSI scenes.

Category	Examples of keywords
Racial groups	African American, Black, White, Biracial, Minority
Racism	Injustice, prejudice, oppressed
Physical features	Weaves, curly, hair, skin
History	Civil Rights, Apartheid, Tuskegee
Geography	South Side, Jamaica
Lineage	Ancestor, elder, descent

Our full list of keywords was entered into the NVivo Text Search tool, which highlighted all instances of these words (including stems) within each interview. In every transcript, LSI scenes were demarcated by interview prompts, marking a shift from one scene to the next. Each interview was trimmed to include only the scenes, unique to each participant, containing one or more racial keywords.^3^ In this process, one scene, *Life Chapters*, was split into smaller sections due to the lengthy responses it elicited, and each section was considered a separate scene. These trimmed “racial narratives” were used for subsequent analysis.

Given that our list of race‐related keywords determined our focus for subsequent analysis, it is important to highlight that researchers' positionality impacted our particular selection of keywords, and in turn, the scope of this study. While our list of keywords and set of racial narratives are not exhaustive, our inclusion of multiple researcher perspectives and broad‐ranging keywords are strengths of the current study, allowing us to capture as much race‐related content as possible.

### 2.6 Qualitative analysis

Racial narratives were organized in order of keyword frequency. Following the steps for thematic analysis outlined by McAdams (2012), the authors started with the interview containing the highest frequency of race‐related keywords, highlighting noteworthy excerpts, and annotating the interview with inferences. The authors met to discuss their impressions, reflecting on how their own experiences shaped what stood out to them in the interview. The authors repeated this process for each subsequent interview, meeting regularly to discuss their impressions, and continuing this process until they started to see recurring themes. These recurring themes were organized into an initial list of categories. To explore how these categories emerged across the broader sample, the authors then assessed a subset of interviews for the presence or absence of each category, refining the categories in this process to develop a final list of themes.

### 2.7 Translating themes into quantitative data

Following Syed and Nelson's (2015) guidance on quantifying qualitative data, a working coding manual was developed to include a description of each theme, inclusion and exclusion rules, examples of excerpts that would be coded as each theme, and negative cases. The full set of 460 racial narratives was then coded for the presence or absence of these themes (*presence = 1*; *absence = 0*). The unit of analysis was defined as each scene, meaning that raters assigned separate codes to each scene within each participant's racial narrative. The themes were divided into two subsets, and a team of two raters coded the narratives for each subset of themes. Raters coded the narratives in eight batches, and interrater reliability was assessed for each batch. Raters met regularly to discuss disagreements, including further discussion of how their own experiences shaped their interpretation of the data. The coding manual was updated after each meeting to reflect a developing mutual perspective on the scope of each theme. The final coding manual is available here: https://osf.io/tr7zv/.

**FIGURE 1:**
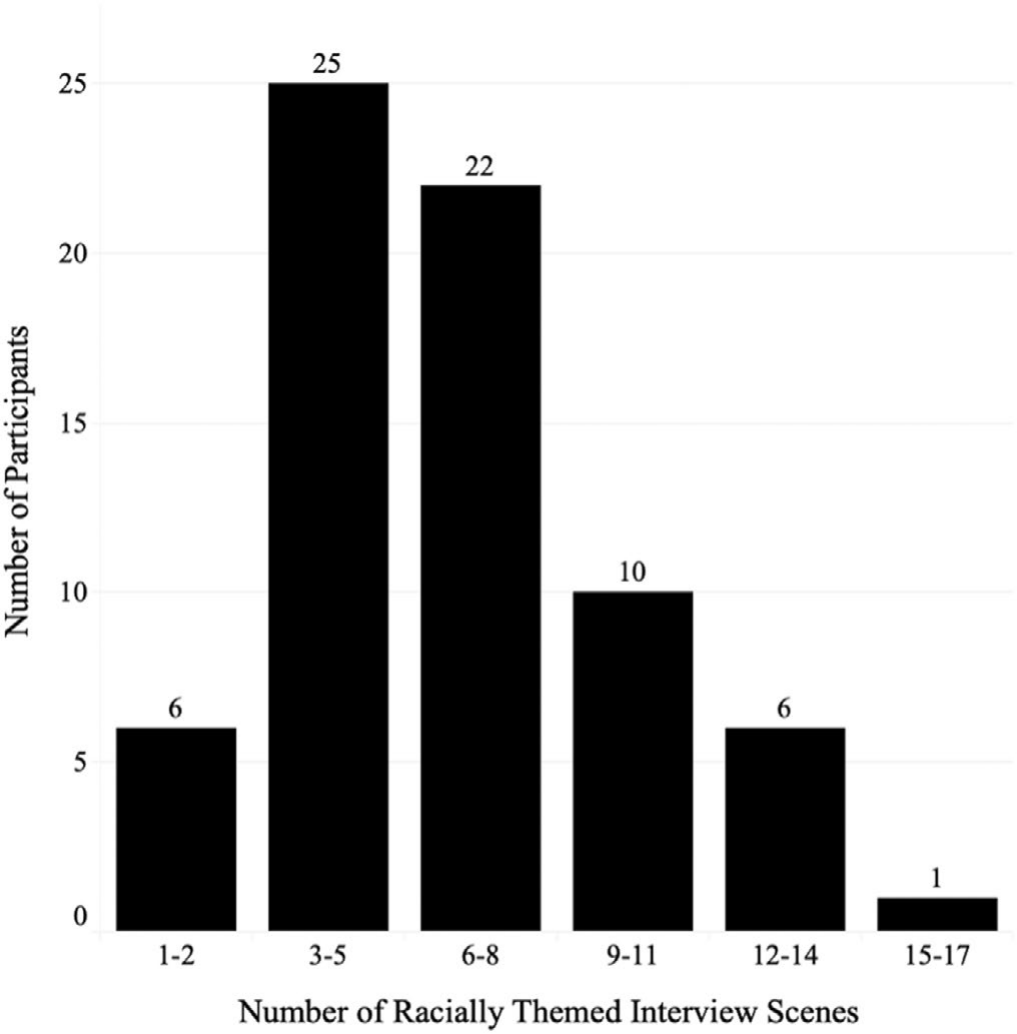
Frequency distribution of racially themed interview scenes.

Each raters' codes were combined into a master spreadsheet. Average codes were calculated across each team, for each data point, and used for subsequent analysis. Overall, frequencies of each theme were calculated by summing codes for each theme. Scene‐level data were aggregated to create composite scores for each theme at the participant level. Since participants differed in the number of total scenes within their racial narratives, a new variable, Number of Racially Themed Scenes, was created to capture this information about the overall salience of race within each participant's life story (Figure 1).

### 2.8 Self‐report measures

Within their battery of self‐report measures, participants completed measures of psychological well‐being, wisdom, and generativity. Psychological well‐being was measured using Ryff's Scales of Psychological Well‐Being (PWB; Ryff & Keyes, 1995), which considers well‐being across six dimensions: autonomy (“my decisions are not usually influenced by what everyone else is doing”), environmental mastery (“I am quite good at managing the many responsibilities of my daily life”), personal growth (“I think it is important to have new experiences that challenge how you think about yourself and the world”), positive relationships (“people would describe me as a giving person, willing to share my time with others”), purpose in life (“some people wander aimlessly through life, but I am not one of them”), and self‐acceptance (“I am quite good at managing the many responsibilities of my daily life”). Participants rated how much they agreed or disagreed with items on a 1–6 scale, ranging from “strongly disagree” to “strongly agree.” Cronbach's alpha for the current sample was 0.92 across all items.

Wisdom was measured through the Self‐Assessed Wisdom Scale (SAWS; Webster, 2003), which considers wisdom as a combination of five dimensions: experience (“I have seen much of the negative side of life”), emotion regulation (“I can regulate my emotions when the situation calls for it”), reminiscence and reflectiveness (“I often recall earlier times in my life to see how I've changed since then”), openness (“I'm very curious about other religions and/or philosophical belief systems”), and humor (“I can make fun of myself to comfort others”). Participants rated how much they agreed or disagreed with items on a 1–6 scale, ranging from “strongly disagree” to “strongly agree.” Cronbach's alpha for the current sample was 0.82 across all items.

Generativity, broadly defined as concern for future generations, was measured through the Loyola Generativity Scale (LGS; McAdams & de St Aubin, 1992), which includes items such as, “I try to pass along the knowledge I have gained through my experiences,” “I have a responsibility to improve the neighborhood in which I live,” and “I have created things that have made an impact on other people.” Participants rated how often the statement applied to them, on a 0–3 scale ranging from “never*”* to “very often or nearly alway*s*.” Cronbach's alpha for the current sample was 0.80 across all items. Descriptive statistics for all self‐report measures are reported in Table 3.

### 2.9 Quantitative analysis

Analyses were conducted in JMP Pro 16. Descriptive statistics were assessed for all variables, and Pearson correlations between variables were examined.

## 3 RESULTS

Our analysis consisted of three steps: (1) exploring the prevalence of racial scenes within a set of life story interviews, (2) identifying salient themes among these narratives, and (3) examining relations between racial narrative themes and psychological measures. We present our results in order of these steps.

### 3.1 Prevalence of racial scenes

Through a broad keyword search for race‐related terms, we identified 460 racially themed interview scenes. All participants referenced race at least once during their Life Story Interview, and the number of scenes in which participants referenced race ranged from 1 to 17 (Figure 1) with an average of 6.57 racially themed scenes. Participants in the lowest range of racially themed scenes tended to reference race indirectly and sparsely, through words such as “wig,” “community,” and “Baptist.” Participants in the higher ranges tended to reference race directly and frequently within each scene, using terms such as “Black,” “African American,” and “racism.”

Of the 460 total racial narrative scenes, the majority fell within the *Key Scenes* and *Life Chapters* sections of the Life Story Interview. This was in part due to the differing lengths of interview sections, with *Key Scenes* containing more scenes (prompts) than any other section, and *Life Chapters* eliciting lengthy responses that were ultimately broken down and counted as multiple scenes.

Overall, 152 scenes (33%) fell within the *Key Scenes* section of the Life Story Interview. Asked to recall a vivid memory from her adult life, for example, Shirley referenced her Black identity while describing what it meant to her family when she walked across the stage to receive her law degree^4^:

It was significant for my mother, it was significant for my uncles who were there and I remember one of my professors…didn't understand why all my family had to be there…but for a Black family…what I didn't say was I was the first person in my entire extended family to even get an undergraduate degree.

Likewise, 118 scenes (26%) fell within the *Life Chapters* section of the interview. For example, Elizabeth describes the racial dynamics that defined her childhood school commute, and how the Black community represented a place of safety for her:

I would take a bus from my house to Halsted. I would get off at Halsted. Take another bus to 79th where there was the end of that line. And then I would get on another bus to go to 87th Street, and then I'd take 87th Street and then go over. Because that way I could stay in the Black community all the way through…

In addition, 92 scenes (20%) fell within the *Personal Ideologies* section. Asked to describe her political and social values, for example, Jada connects the question to her racial identity:

Obviously, as a African‐American, I'm concerned with issues of racism and prejudice…you know, how far can a light skinned African‐American person get beyond what a dark skinned African‐American person could get?

Likewise, when asked to describe his religious and ethical beliefs, Robert offers historical context for his religious upbringing: “I believe that because of the history of slavery in my family, because they ended up on a cotton plantation in 1863 at the end of slavery, I am a Baptist.”

A noteworthy portion of scenes also fell into the *Future Script* and *Challenges* sections of the Life Story Interview, and 47 scenes (10%) fell within the *Future Script* section. Prompted to describe a project she is currently working toward, for example, Elizabeth described her goal of working with African American students to, “explore the African past so that they can take pride in…who their ancestors were and the accomplishments that they achieved throughout history…” Likewise, 40 scenes (9%) fell within the *Challenges* section. Asked to describe her greatest challenge, for example, Ebony stated, “my challenge has been to be a single mother and to raise two Black boys in the City of Chicago.”

Only 8 scenes (2%) fell within the *Reflection* section, and 3 scenes (1%) fell within the Life Themes section of the interview.

### 3.2 Racial narrative themes

Through an iterative process of reading racial narratives and keeping track of themes, we identified seven overarching racial narrative themes: *Black Cultural Identity, Community of Care, Systemic Racism*, *Activism*, *Racial Reckoning*, *Multiculturalism*, and *Encounter with Racism*. We present these themes, along with an exemplary quote, in Table 2.

**TABLE 2:** Salient racial narrative themes.

Theme	Description	Example
Black Cultural Identity	Expressly identifying as Black/African American	“I have a lot of pride in being Black and want to lift my people up. I just feel like culturally, Black people as a race need to love each other more, cause we've been through a lot”
Community of Care	Sense of being part of a larger community of support	“We used to sit and listen to the stories of my great aunts and my grandmother, my great grandmother, my mother…we have six living generations”
Systemic Racism	Reference to the broader context of racism	“Where I grew up there was a street called South Parkway…it was a dividing line…on the west side was where people who were considered poorer or disadvantaged lived which is where I lived”
Activism	Motivation to contribute to the larger world	“I saw something that I didn't like pertaining to how Black children were being expelled in school…and I said I want to do something about it. I became an equal justice works fellow”
Racial Reckoning	Moment of grappling with racial identity	“People would ask me well, aren't you afraid, you're going to go to Vietnam…I wasn't afraid because the balance of just Black on Black crime, and the riots…I was in more of a danger zone there than going to Vietnam…”
Multiculturalism	Worldview that celebrates diversity	“I love…how the Native Americans and the Asians—how much trust they put in the wisdom of elders. And the African cultures too. Everything went through the elders”
Encounter with Racism	Vivid memory of dealing with racism	“I remember once my dad stopping to get gas…when my dad asked if we could use the bathroom, he pointed us to an outhouse”

#### 3.2.1 Black cultural identity

“Black people as a race need to love each other more…” Originally developed to capture a sense of cultural pride and collective identity, this theme was broadened to include any instance of expressly identifying as Black or African American. Speaking for the collective, for example, Kadeem expressed, “we Black folks…we been praying to God for I don't know how long.” Likewise, Jamal made it clear that he felt a sense of connection to his African American identity: “I see these things on the news sometimes like where, particularly in my community, and what I mean by my community, African American community…where there's so much…too much violence.”

Other participants seemed to mention race incidentally. However, we interpreted any choice to name race as a meaningful expression of racial identity, given that participants brought up race without any prompting to do so. For example, describing a project she is working on, Tamika states that she is, “writing a story about African American children about a regular life, you know…him living with his aunt and his sister, being the youngest one.” Although she does not elaborate on the detail that she is writing a story about *African American* children, her choice to include this detail at all suggests that race is relevant to her narrative identity.

Sometimes, participants referred to racial identity more implicitly, such as through racialized language, arts and culture references, descriptions of physical features, and references to countries with large Black populations. We captured all of these implicit references within this theme. Describing how music at Baptist churches has changed over time, for example, Alicia stated, “a lot of the songs sound like the songs that you would sing at a dance…not at a church service. And they're really hippity hop, and they call it gospel rap.” Likewise, describing her husband's family, Chloe reflected, “okay, well his mother was considered very, they were like creoles, and I'm dark, they were creoles, and back then that was a big thing.”

#### 3.2.2 Community of care

“We have six living generations…” Participants also conveyed a sense of being part of a larger community of support. We interpreted “community” broadly, capturing a range of contexts such as family systems, schools, neighborhood environments, and church communities, which highlighted a sense of care and belonging. For example, Charisse, who was raised in a multigenerational household, reminisced, “we used to sit and listen to the stories of my great aunts and my grandmother, my great grandmother, my mother…we have six living generations.” Likewise, describing the community where she grew up, Naima recalled:

Your neighborhood is comprised of everybody in the Black community…we had a policeman that lived on the first floor. My teachers lived, you know, in the neighborhood…I really felt like a part of everybody around

Speaking of her church community with a similar sense of belonging, Jodi reflected, “I grew up in what is called a sanctified church, a branch probably off of Baptist, with the singing and the shouting and the tambourines and so forth.”

#### 3.2.3 Activism

“I want to do something about it.” While *Community of Care* focused on participants' immediate support systems and often came up in the context of childhood, *Activism* reflected participants' desire to give back to their community. This often came up in the context of professional pursuits. Reflecting on her motivation for becoming a lawyer, for example, Alicia stated

The reason was because of the era in which I grew up, the civil rights era. And looking at what things were like where I grew up…I saw something that I didn't like pertaining to…Black children who were being expelled in school in that particular community and I said I want to do something about it. I became an Equal Justice Works Fellow.

Likewise, Belinda majored in Education, Social Studies, and History in college, because she wanted to teach African American history so that her students would, “know the greatness where they came from.”

Importantly, *Activism* included both small and large‐scale efforts to contribute to a better world. For example, Camryn organized a community gathering at her church: “I had an adopted Jewish mother who would invite me to seders. And so I was like we African American people need something like this. And so I did one last year at the church.”

#### 3.2.4 Multiculturalism

“We need to build bridges.” While many participants expressed a connection to their Black/African American identity, some expressed an appreciation for other racial and cultural groups. Describing the environment of the new high school to which she transferred, for example, Mercy reflected, “I was able to interact with people of all races, you know, Hispanics, you know, whites, people from different ethnic groups…” She added that this gave her, “a chance to kind of explore different types of personalities and, and different cultures.”

In addition to appreciating experiences with people of different cultures, participants also shared worldviews that emphasized a sense of shared humanity. Reflecting on her internal shift toward this worldview, for example, Mariah stated, “the focus of my life is not on race and racial differences. It's on the things that bring us together…us all being people who share this earth together.”

Expressions of such worldviews extended beyond race. Emphasizing a sense of unity across religious groups, for example, Shonda stated:

I'd say that religion is something that you take upon yourself, whether you're Catholic, Greek Orthodox, Jewish, Baptist, Pentecostal, whatever. I believe, and this is my personal belief, that if you put all of them gods up there, they'd be falling out the sky. So cause there's not enough room. So I believe that basically we all worship the same person, but we all call it by a different name.

#### 3.2.5 Systemic racism

“It was a dividing line…” References to racism were also common in these racial narratives and spanned a broad spectrum from direct experiences of racial violence to descriptions of inequity to memories of historic events. Noting a qualitative difference between vivid memories of racism and references to the broader context of living in a racist society, we coded separately for *Encounters with Racism* and *Systemic Racism*.

When referencing broader societal conditions, participants sometimes made the connection to racism explicit. Describing differences between the educational resources available to Black and white students during his childhood, for example, Denzel recalled, “I lived one block away from an all white school which was new, which had a cafeteria, which had a indoor gymnasium. School I went to had four classrooms.” In other cases, participants did not explicitly mention racism, and yet the conditions they described–neighborhood divisions, protests, being the “first” or “only” Black person at their workplace–were clearly downstream effects of racism. Thus, we captured both direct and indirect references to racism within this theme. For example, Nina described how, “the ‘70s was about getting minorities into college,” implicitly referencing the educational barriers for students like her. She then elaborates:

[College] had never been something that I had really ever thought about. You are thinking…the family does not have money, you do not really have a guide, no one else before you has gone, so you do not really have anyone that is pushing for it…

Although Nina does not name racism explicitly, she describes conditions that are a result of racism: systemic barriers that prevented her family and community members from attending college before her and having the money to send her to college.

#### 3.2.6 Encounter with racism

“He pointed us to an outhouse.” In contrast to *Systemic Racism*, descriptions of overt racism—vivid memories of experiencing or witnessing racism—were less frequent throughout our racial narratives. Still, these moments were striking for the imprint they left on participants. For example, Leena described:

I remember once my dad stopping to get gas, and we had to use the bathroom, and as the guy was pumping the gas…when my dad asked if the kid—if we could use the bathroom, he pointed us to an outhouse.

While most encounters with racism involved interpersonal interactions, some participants placed greater emphasis on the broader historical context, while still recounting a vivid memory of racism. For example, Joy recalled:

It was announced that the schools were integrated…and there was a lot of rioting in the hallway when we began to change class, there was fights and this and that. So my friend and her friends came and said we've got to get you out of here, they're going to attack you. And I said no, we're not going to run…and she kept saying no, you've got to get out of here. I said no…I have to stay here and I have to deal with the consequences. And that day was the day that they all stood up with me.

#### 3.2.7 Racial reckoning

“Is all white people bad or are some white people good?” The racial narratives in our data set were primarily descriptive: most of the time, participants brought up race while recounting some experience or aspect of their life story, without reflecting on race directly. Some narratives, however, contained this extra layer reflecting on race, racial identity, or racism. We captured these narratives within the theme of *Racial Reckoning*. Reflecting on the racial dynamics at the women's advocacy organization where she and another woman were the first African American employees, Adrienne recalls:

we had issues come up around that, you know, some ways that we were similar and in some ways that they didn't understand us and we didn't understand them…that was kind of my first experience in sitting down with people and having sort of sensitivity sessions, you know.

Likewise, reflecting on his reasons for joining the army, Joseph recalled:

People would ask me well, well, aren't you afraid, you're going to go to Vietnam, and, and I wasn't afraid because it—the balance of just Black on Black crime, and the riots…I was in more of a danger zone there than going to Vietnam, you know.

### 3.3 Translating qualitative themes into quantitative data

Upon identifying these racial narrative themes, we “coded” each scene, in two teams, for the presence or absence of each theme. Importantly, these themes were interrelated and not mutually exclusive. Overall interrater reliability ranged from good to excellent: *M*_𝜅_ = 0.70 and *M*_% agreement_ = 87 for team one; *M*_𝜅_ = 0.76 and *M*_% agreement_ = 88 for team two. Interrater reliability for individual themes ranged from fair to excellent: for *Black Cultural Identity*, 𝜅 = 0.78 and % agreement = 89; for *Community of Care*, *𝜅* = 0.54 and % agreement = 77; for *Systemic Racism*, 𝜅 = 0.64 and % agreement = 83; for *Activism*, 𝜅 = 0.65 and % agreement = 87; for *Racial Reckoning*, 𝜅 = 0.56 and % agreement = 86; for *Multiculturalism*, 𝜅 = 0.70 and % agreement = 94; for *Encounter with Racism*, 𝜅 = 0.71 and % agreement = 96. Table 3 presents descriptive statistics, including the mean, standard deviation, minimum, maximum, and range for each study variable.

**TABLE 3:** Descriptive statistics for study variables.

	*M*	*SD*	Min	Max	Range
*Narrative variables*					
Number of racially themed scenes	6.57	3.43	1	17	16
Community of care	3.21	1.88	0	9.5	9.5
Black cultural identity	3.24	2.97	0	15	15
Multiculturalism	0.72	0.82	0	3	3
Activism	1.63	1.86	0	8	8
Encounter with racism	0.49	0.83	0	4.5	4.5
Systemic racism	2.5	2.54	0	14	14
Racial reckoning	1.44	2.2	0	12	12
*Self‐report measures*					
Psychological well‐being	5.12	0.59	2.76	5.90	3.14
Wisdom	4.64	0.59	3.05	5.85	2.80
Generativity	2.29	0.42	0.85	2.9	2.05

**FIGURE 2:**
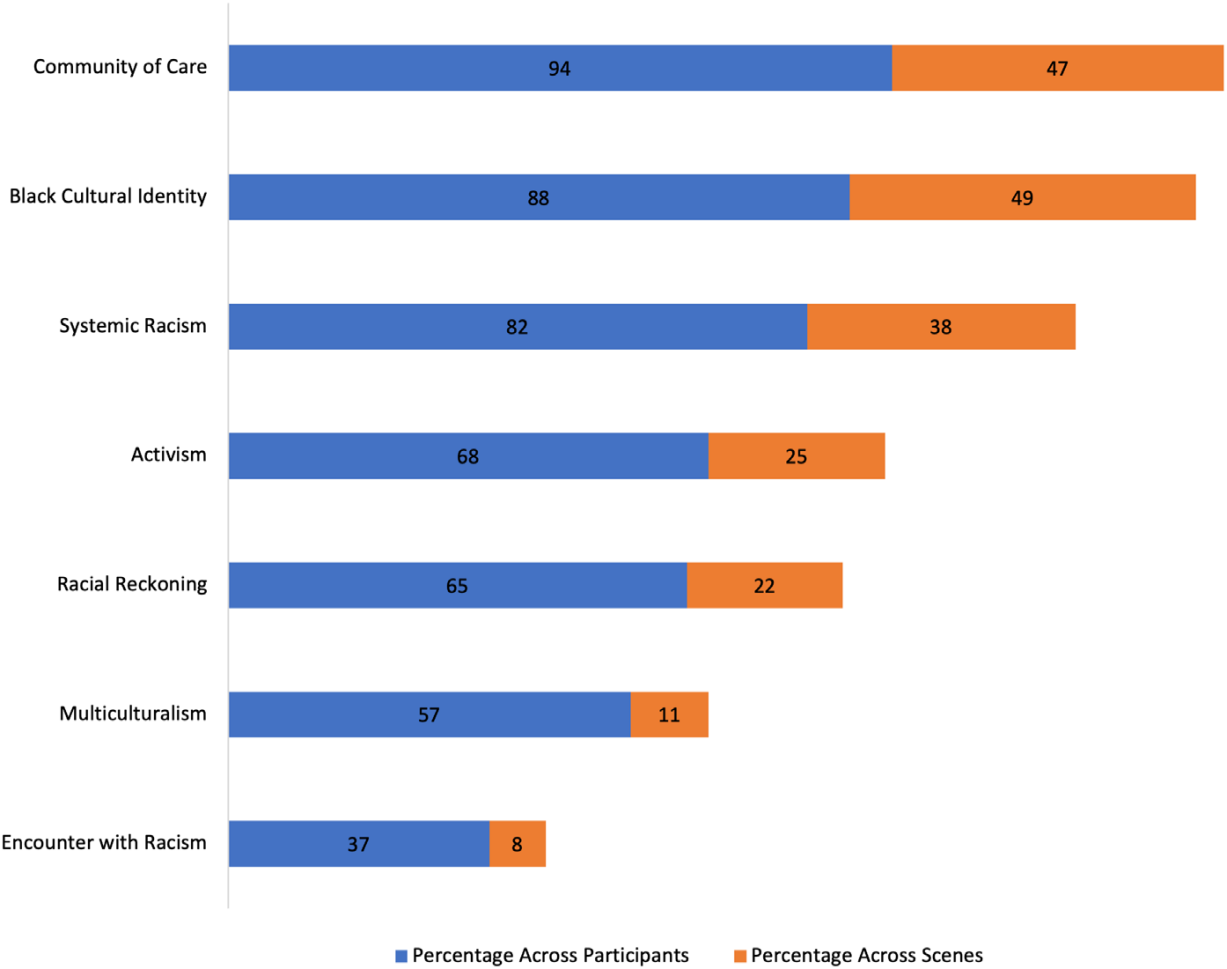
Prevalence of racial narrative themes.

Most narrative themes spanned a wide range of scores and were skewed toward the lower end of the distribution. *Multiculturalism* and *Encounter with Racism* spanned narrower ranges. These themes were less common overall: several participants did not include these themes in their racial narratives, and those who did included them relatively infrequently. These differences in the range of our narrative themes may be due to differences in nature of each theme: *Black Cultural Identity*, *Community of Care*, and *Systemic Racism*, in particular, were broader constructs that encompassed a wide variety of responses, while *Multiculturalism* and *Encounter with Racism* were narrower constructs and therefore captured fewer and a smaller range of responses. Complimenting these descriptive statistics, we also examined the prevalence of each theme across participants and across all scenes (Figure 2). *Community of Care* and *Black Cultural Identity* were the most frequently occurring themes, with *Community of Care* represented across 94% of participants and within 47% of total scenes coded and *Black Cultural Identity* represented across 88% of participants and within 49% of total scenes coded. In comparison, *Multiculturalism* was less common, represented across 57% of participants and within 11% of total scenes coded. Likewise, *Encounter with Racism* was the least frequently occurring theme, represented across 37% of participants and within 8% of total scenes coded.

Finally, most of our narrative variables were significantly correlated with each other (Table 4). All themes were moderately to highly correlated with overall number of racially themed scenes (*r* = 0.42 to *r* = 0.76): in other words, talking about race in a wider selection of Life Story Interview scenes corresponded to higher levels of racial narrative themes. This makes sense, given that scenes were selected for their racial content and themes were derived directly from these scenes. Furthermore, most themes were moderately correlated with each other (*r* = 0.20 to *r* = 0.51), and some themes were highly correlated with each other (*r* = 0.66 to *r* = 0.85). For example, *Black Cultural Identity, Encounter with Racism, Systemic Racism* and *Racial Reckoning* were highly intercorrelated, which makes sense, given that participants often referenced their Black/African American identity when highlighting incidents of racism, referencing broader systems of oppression, and reckoning with race. Furthermore, stories about encounters with racism were likely to contain references to broader systems at play and reflections on the experience. And when participants reckoned with race, for the most part, this meant reflecting on some aspect of racism. Likewise, *Activism* was also highly correlated with *Black Cultural Identity, Systemic Racism*, and *Racial Reckoning*, which highlights a noteworthy relationship between participants' tendency to incorporate race and racism into their narrative identities and their motivation to contribute to the broader world.

**TABLE 4:** Correlations among study variables.

	PWB	WIS	GEN	NRTS	COMM	BCI	MULT	ACT	ENCR	SYSR
PWB	—									
WIS	0.47***	—								
GEN	0.45***	0.47***	—							
NRTS	0.18	0.26*	0.37**	—						
COMM	0.08	0.12	0.23^†^	0.70***	—					
BCI	0.13	0.24*	0.33**	0.75***	0.43***	—				
MULT	0.16	0.30*	0.37**	0.49***	0.29*	0.46***	—			
ACT	0.19	0.28*	0.37**	0.76***	0.36**	0.70***	0.47***	—		
ENCR	0.08	0.04	0.18	0.42***	0.26*	0.66***	0.20	0.43***	—	
SYSR	0.11	0.18	0.31*	0.67***	0.41***	0.85***	0.51***	0.69***	0.73***	—
RACR	0.13	0.23	0.28*	0.58***	0.34***	0.81***	0.45***	0.60***	0.76***	0.82***

Abbreviations: ACT, activism; BCI, Black cultural identity; COMM, Community of Care; ENCR, encounter with racism; GEN, generativity; MULT, multiculturalism; NRTS, number of racially themed scenes; PWB, psychological well‐being; RACR, racial reckoning; SYSR, systemic racism; WIS, wisdom.

^†^ *p* = 0.06.

* *p* < 0.05;

** *p* < 0.01;

*** *p* < 0.001.

### 3.4 Associations with psychological measures

The final aim of this study was to examine correlations between racial narrative variables (including racial narrative themes and Number of Racially Themed Scenes) and three measures of psychological health: psychological well‐being, wisdom, and generativity. Table 4 includes these correlational results, which suggest that the salience of race within the Life Story Interview is associated with wisdom and generativity. Specifically, generativity showed significant positive correlations with the *Number of Racially Themed Scenes* (*r* = 0.37), *Black Cultural Identity* (*r* = 0.33), *Multiculturalism* (*r* = 0.37), *Activism* (*r* = 0.37), *Systemic Racism* (*r* = 0.31), and *Racial Reckoning* (*r* = 0.28). Wisdom showed significant positive correlations with *Number of Racially Themed Scenes* (*r* = 0.26), *Black Cultural Identity* (*r* = 0.24), *Multiculturalism* (*r* = 0.30), and *Activism* (*r* = 0.28). Although wisdom and generativity were both significantly correlated with well‐being, none of the racial narrative variables showed significant correlations with well‐being.

## 4 DISCUSSION

The purpose of this study was to explore the role of race within the broader narrative identity of Black Americans in midlife. While telling their life stories, 100% of participants referenced race, and the number of interview scenes in which participants referenced race ranged from 1 to 17, highlighting both the overall salience of race for participants as they narrated their life stories and the natural variance in the extent to which participants brought up race while telling their stories. We use the term *salience* rather than *centrality*, in line with Sellers et al.'s (1998) MMRI, to highlight that this interview represents a particular situational context. Participants were asked to tell their life stories without any prompting to speak specifically about race, and in most cases, participants shared their life stories with a white interviewer. It is likely that a different situation—for example, being prompted to speak about race and/or speaking with Black interviewers only—would have resulted in different levels of racial discourse throughout the Life Story Interview. However, it is not entirely surprising that race‐related content was mentioned by all participants in our sample, given the cultural context of racism in the United States and the cohort effect of growing up during the highly racially salient civil rights period.

The interview sections themselves, which center around different aspects of a person's narrative identity, represent small shifts in the situational context of the interview, and we found differences in the overall percentage of race‐related scenes by interview section. For the most part, these differences are proportional to the length of each interview section. Notably, the Life Chapters section was overrepresented among race‐related scenes, and this is likely due to the nature of the prompt, whereas most other prompts focused on a specific moment or area of life, this prompt invited participants to imagine their lives as a book or a novel and freely describe each chapter. If race played an important role in any part of a person's life story, it was thus likely to come up in this section. Likewise, the comparatively lower frequency of racial content in other sections may reflect the nature of these specific prompts more than the role of race within the person's broader narrative identity.

That said, in some cases, race appeared as a throughline within participants' interviews, which was noteworthy, given that participants were not prompted to speak about race. We highlight one such case below as a starting point for discussion of our qualitative results.

### 4.1 James's story

James, a 55‐year‐old man, begins his life story by describing the racial violence that characterized his school commutes in the south during the Civil Rights Movement: “we had to go to school through a all‐white community, and they were taking shots with .22s at us.” At the same time, he recalls how his third‐grade teacher cultivated a caring space that helped him develop inner resources amidst this racial violence:

She told us that being Black children we will be faced with some severe and unfair things that go on in this world. And she said, you will either break through them or let that situation destroy you. She said, so what you will do in my room is learn a series of poems of your choice…and each day that I stop you…you'd better be able and ready to recite that powerful piece that encourages you when you're going through troubled times.

In the sixth grade, James moved to Michigan, where he experienced an integrated classroom for the first time. On the one hand, he recalls positive encounters with white people that lead him to question, “is all white people bad or are some white people good?” On the other hand, he recalls growing increasingly aware of the racial disparities at his school:

Why is it that I'm in an English class or a math class with nothing but black kids and everybody's cracking jokes and not taking their education serious and I walk down the hall and there's nothing but white kids in there that is doing great things in the community?

After high school, James joins the Marine Corps, where his commanding officer encourages him to think about college. At this point, James realizes that no teacher, coach, or counselor ever asked him about college:

It didn't dawn on me what the big picture was…that I was being programmed to wash somebody's dishes, to clean somebody's car, to sweep somebody's floor or go into somebody's military and put my life on the line. That's all I was good for.

Considering his options, James applies to a college near where his mom lives, so he could be around to help take care of her. After college, he goes on to become a teacher, and later, a principal and superintendent.

James's life story also included reflections on his religious or spiritual background: “I've always been spiritual, but I respect other religions…Buddhism, Hinduism, whatever, I'm happy for you. But in my spirituality, I believe in the Supreme Being…and I ask only that he continue to give me the ability to serve others.” Over the course of the interview, James shares his deeper motivation for serving others, rooted in his racial experience:

No matter what I do in life, my goal is to raise every Black man and boy level of consciousness so they could understand what they're against, you know, and what they are facing in this society.

In sum, the following features of James's story emerged, to varying degrees, throughout the rest of the life stories in our data set: references to both the broader context of racism (*Systemic Racism*) and vivid memories of dealing with racism (*Encounter with Racism*), a strong sense of Black/African American identity (*Black Cultural Identity*), a sense of being part of larger community of support (*Community of Care*), a motivation to contribute to the larger world (*Activism*), a worldview that celebrates other cultures (*Multiculturalism*), and a sense of grappling with issues related to race (*Racial Reckoning*).

These qualitative results fall in line with existing frameworks for understanding racism and racial identity development. For example, *Systemic Racism* and *Encounter with Racism* parallel structural and interpersonal interpretations of racism. Furthermore, *Systemic Racism* occurred more frequently across our data set than *Encounter with Racism*, exemplifying a phenomenon that racial identity researchers have previously highlighted: individuals are often hesitant to explicitly name experiences of racism, and this hesitance itself reflects a cultural norm (Rogers et al., 2022). Yet racism often operates covertly through systems rather than overtly through interpersonal interactions, and our results suggest that individuals may be more comfortable referencing covert racism while telling their life stories.

Our life story data also reflect the notion of racial socialization: that as Black children grow up, community members can help reduce the negative impacts of racism by raising awareness about structural inequality, promoting critical consciousness, and nurturing a strong sense of identity (Umami‐Taylor & Hill, 2020). For example, James's third‐grade teacher instructed her students about the inequalities they would face as Black children and equipped them with tools to face this harsh reality. In a review of the literature on parents’ ethnic‐racial socialization, Hughes et al. (2006) identified the following themes, which parallel themes in our set of racial narratives: (1) *cultural socialization*, which includes instilling racial pride as captured within *Black Cultural Identity*, (2) *preparation for bias*, which involves raising awareness about racial barriers and racism, as captured within *Community of Care* and *Activism*, (3) *promotion of mistrust*, which emphasizes the need for caution in interracial interactions, and (4) *egalitarianism*, which highlights equality among ethnic‐racial groups, as captured within *Multiculturalism*.

In some cases, our racial narratives and corresponding themes also mapped onto Cross's (1971) stages of Nigrescence. At the start of his story, for example, James describes experiences of racial violence during the Civil Rights Movement (*Encounter/Encounter with Racism*), which prompts him to grapple with his racial identity (*Immersion‐Emersion/Racial Reckoning*). His racial consciousness is deepened when his family moves to Michigan and he attends an integrated school for the first time, where he observes differences in opportunities afforded to Black and white students (*Internalization/Systemic Racism*). Over time, James's racial consciousness motivates his goals to improve the educational system for all kids and to, “raise every Black man and [boy's] level of consciousness so they could understand…what they are facing in this society” (*Internalization Commitment/Black Cultural Identity/Activism)*.

Likewise, our racial narrative themes also reflect elements of the MMRI (Sellers et al., 1998). For example, the MMRI includes the dimension of *Racial Ideology*, a person's philosophies about how members of their racial group should relate to society. These philosophies include nationalist and humanist perspectives. The nationalist perspective highlights the unique history, culture, and accomplishments of African Americans, as captured within *Black Cultural Identity*, while the humanist philosophy emphasizes similarities among all human beings, as captured within *Multiculturalism* (Sellers et al., 1998).

Given considerable alignment between our qualitative results and existing models of racial identity, the current study straddles the line between racial identity and narrative identity research. In both fields, scholars are ultimately interested in how dimensions of their respective constructs relate to psychological outcomes. Within the field of racial identity, most studies examining psychological outcomes rely on self‐report measures of racial identity (e.g., Chew, 2015; Elmore et al., 2012 ; Johnson & Carter, 2020; Lewis et al., 2018; Willis et al., 2021). By capturing racial identity through a set of narrative themes, the current study expands the literature on how racial identity relates to psychological outcomes. Likewise, by identifying race‐related narrative themes, we also highlight how race is important to the study of narrative identity among Black Americans, and our qualitative results are bolstered by a quantitative exploration of relations between race‐related narrative themes and psychological outcomes of well‐being, generativity, and wisdom.

We found that higher levels of racial narrative variables correlated with higher levels of generativity and wisdom but did not correlate with psychological well‐being. While these correlational results should be interpreted with our relatively small sample size (*N* = 70) in mind, our findings related to generativity, in particular, build on previous research at the intersection of race and narrative identity.

Black Americans tend to show higher levels of concern for and commitment to the next generation than their white counterparts (e.g., Hart et al., 2001; Newton & Baltys, 2014) and report greater numbers of generative acts (McAdams, 2013) as well as a greater emphasis on community when considering the type of legacy they wish to leave behind (Newton & Jones, 2016). Furthermore, Black Americans score higher on related measures of social involvement, including political engagement, civic engagement, public service motivation, and religious engagement (Jones & McAdams, 2013). With respect to generativity and narrative identity, McAdams (2013) characterized the generative life in terms of five narrative themes—*early advantage, sensitivity to suffering, moral steadfastness, redemption sequences*, and *prosocial goals*—and McAdams and Guo (2015) found that Black participants told life stories with increased themes of *moral steadfastness* and *prosocial goals*.

While these studies offer compounding evidence for racial differences in generativity, it is important to consider why these differences exist: Fabius (2016) highlights the unique historical context and experiences of racial oppression that have shaped African American elders as well as the importance of multigenerational relationships as a source of resilience, strength, and hope among African American families throughout history (Waites, 2009). Likewise, storytelling has served as an important way for African American elders to pass on important wisdom, values, and methods of resilience to younger generations (Fabius, 2016). Generativity and wisdom are, in other words, a means by which older generations of Black Americans have helped younger generations navigate and survive within systems of racial oppression. There is thus an adaptive quality to generativity and wisdom for Black Americans that does not exist in the same way for members of other racial groups.

It is possible that the Life Story Interview may have invited expressions of generativity and wisdom. This is to say that for some participants, the interview itself may have served as an opportunity to pass on a story of the self to the next generation, as a way of providing guidance, instruction, or enlightenment for that generation. In a sense, some participants may have implicitly taken on the role of a teacher, with the aim of contributing responsibly to society by sharing experiences related to race with mostly young, white interviewers.

The nonsignificant relationship between racial narrative variables and well‐being is also worth exploring, given general interest among psychologists in this outcome measure and evidence of a relationship between self‐report measures of racial and ethnic identity and positive mental health outcomes (e.g., Chew, 2015; Neblett et al., 2012; Rivas‐Drake et al., 2014; Smith & Silva, 2011; Yip et al., 2019). One possibility is that our particular measure of racial identity—the extent to which individuals talk about race, without prompting, while telling their life stories—simply has no bearing on a person's well‐being. Another possibility is that the nature of racial discourse within the Life Story Interview renders relations with well‐being complex. For example, Syed et al. (2013) found that ethnic identity exploration is comprised of two dimensions, participation and search, which show opposing relations to well‐being. As our racial narratives also reflect a process of identity exploration, further analysis might uncover dimensions beyond those we have identified in the current study that differentially relate to well‐being.

Finally, it is likely that our findings are particular to the developmental and social context of our participants. For example, Smith and Silva (2011) found that associations between ethnic identity and well‐being were stronger for younger adults than adults over the age of 40. Given that midlife marks a shift toward caring for future generations, the age group of our sample may have contributed to relations between racial narrative variables and generativity. Relatedly, marginalizing societal structures makes traditional pathways to a “good life” less accessible to some groups than others (Bauer, 2021; Syed & McLean, 2022). Marginalization may also lead to alternate conceptions of a good life (Syed & McLean, 2022). It is possible that generativity and wisdom are more closely aligned with what good life has come to mean for the middle‐aged Black Americans in our sample than traditional measures of well‐being.

## 5 CONCLUSIONS AND FUTURE DIRECTIONS

In the early stages of this study, the first author sought guidance from a racial identity scholar who asked, “why are you doing this project?” The question was both personal and practical. On a personal level, it invited the first author to consider what was drawing her, as a non‐Black person, to this study of Black identity. On a practical level, it called into question the value of studying race within interviews in which participants were not asked about race. Without a doubt, asking questions about race would have allowed for a deeper investigation of the role of race within the life stories of Black Americans. While the setup for the current study is not ideal for this reason, it also offers an example of how to analyze existing data through a modern cultural lens. Specifically, the original Foley Longitudinal Study of Adulthood conceived of race as a fixed demographic category, and the relatively even distribution of Black and white participants in the study was intended for investigation of racial differences in narrative identity and self‐report variables. The current analysis conceives of race as a culturally defined construct, which permeates individual lives in varying ways. To explore this variation, the current analysis builds on strengths of the original study—the inclusion of 73 Black participants and collection of rich narrative and self‐report data—demonstrating how larger narrative accounts such as life stories can be analyzed for specific thematic content, such as scenes relating to race. In doing so, we offer a framework by which future studies may analyze existing narrative data through evolving cultural frames. While we pilot these steps with a sample of middle‐aged Black Americans who grew up during the civil rights era, future studies might apply a similar approach toward narrative data from participants today, or participants of different generations and racial‐ethnic minority groups.

In summary, the current study makes three important contributions. First, we introduce a novel approach for narrative identity research, highlighting how life story interviews can be analyzed for specific thematic content, even when this content is not directly prompted. This approach may be especially useful when working with existing narrative data, as noted above. Second, we offer insight into racial identity from the vantage point of a person's broader narrative identity. Given the extent to which participants in our study talked about race without prompting, our findings suggest that race plays an important role in the life stories of Black Americans, and researchers should keep this in mind when designing future interview protocols to elicit narrative identity. Third, we also found variation in the extent to which race was salient for individuals in our data set, with higher levels of racial narrative themes corresponding to higher levels of generativity and wisdom. While we highlight the social and developmental context that may underly these associations, these constructs were not originally conceived in connection to racial identity, and the specific meaning and quality of generativity and wisdom for Black Americans, in the context of their racial experience, is an area for future exploration.

## AUTHOR CONTRIBUTIONS

Ananya Mayukha and Dan P. McAdams developed the idea for the current analysis. Ananya Mayukha led data preparation and analysis and wrote the manuscript. Ambar Guzman and Sirin Jitklongsub contributed to coding, analysis, and interpretation of results. Dan P. McAdams oversaw original data collection and provided research mentorship and editorial support.

## FUNDING INFORMATION

This research was supported by the Foley Family Foundation.

## CONFLICT OF INTEREST STATEMENT

The authors declare no potential conflicts of interest regarding the research, authorship, and/or publication of this article.

## ETHICS STATEMENT

The Foley Longitudinal Study of Adulthood, on which the current analysis is based, was approved by the Northwestern University Institutional Review Board (IRB) under the protocol ID STU00001801.

## Endnotes

^1^
See Tashakkori et al. (2012) for an explanation of different types of mixed method designs in psychological research. What characterizes a *sequential* design is that each phase of the study depends on the results of the previous phase.

^2^
Life Story Interviews of participants who selected their race/ethnicity as “Interracial” and “Other” contained explicit references to Black identity and were therefore included in this analysis.

^3^
The first author reviewed all instances of keywords and excluded any instances in which words occurred as double meanings unrelated to race, without an additional relevant keyword in the same passage.

^4^
When quoting participants, we use pseudonyms and edit excerpts for brevity. While qualitative data are not publicly available, they are available upon request, and steps to request access are available on the OSF page for this project: https://osf.io/tr7zv/.
